# The Pharmacological Targets and Clinical Evidence of Natural Products With Anti-hepatic Inflammatory Properties

**DOI:** 10.3389/fphar.2018.00455

**Published:** 2018-06-05

**Authors:** Jinghua Peng

**Affiliations:** ^1^Institute of Liver Diseases, Shuguang Hospital Affiliated to Shanghai University of Traditional Chinese Medicine, Shanghai, China; ^2^Shanghai Key Laboratory of Traditional Chinese Clinical Medicine, Shanghai, China

**Keywords:** hepatic inflammation, cytokine, chemokine, inflammasome, toll like receptor, nuclear factor-kappa B, mitogen-activated protein kinases, reactive oxygen species

## Abstract

Inflammation contributes heavily to the pathogenesis of liver fibrosis, cirrhosis, and even hepatocellular carcinoma. Inflammation is probably a promising target for treatment of liver diseases. The natural products are considered as the potential source of new drug discovery and their pharmacological effects on hepatic inflammation have been widely reported. In this review, the natural products with anti-hepatic inflammatory properties are summarized based on their pharmacological effects and mechanisms, which are related to the suppression on the inflammation mediators including cytokines and chemokines, pattern recognition receptors, the activated transcriptional factors, and the potential regulatory factors. The clinical evidence is also summarized.

## Introduction

Hepatic inflammation can be triggered by microbial infection, metabolic disorders, or exposure to drugs and toxic substances (Strazzabosco et al., 2005) and almost exists in every form of liver diseases. The acute hepatitis is usually short and self-limited, while the chronic is characterized with continuing inflammation, tissue injury, and healing. During chronic hepatitis, hepatic stellate cells (HSCs), the fibrogenic cells in the liver, are activated and initiate collagen deposition, which ultimately cause liver fibrosis and cirrhosis. Hence, targeting hepatic inflammation is an important strategy to block the progression to the end state of liver disease.

**Figure 1:**
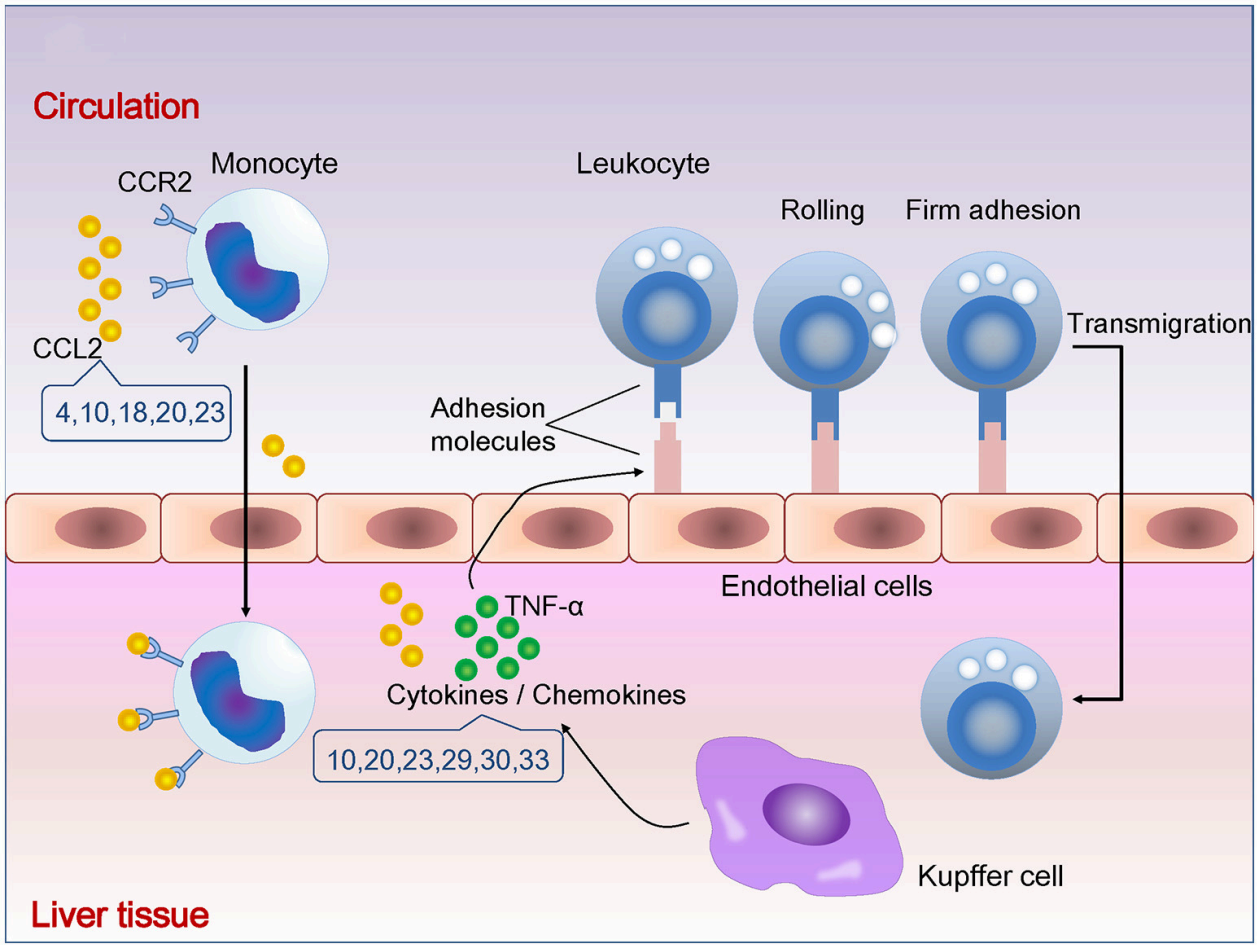
Natural compounds suppressing cytokines/chemokines and leukocytes infiltration. The number indicates compounds described in Table 1. CCR2, C-C chemokine receptor 2; CCL2, C-C chemokine ligand 2; TNF-α, tumor necrosis factor alpha.

**Figure 2:**
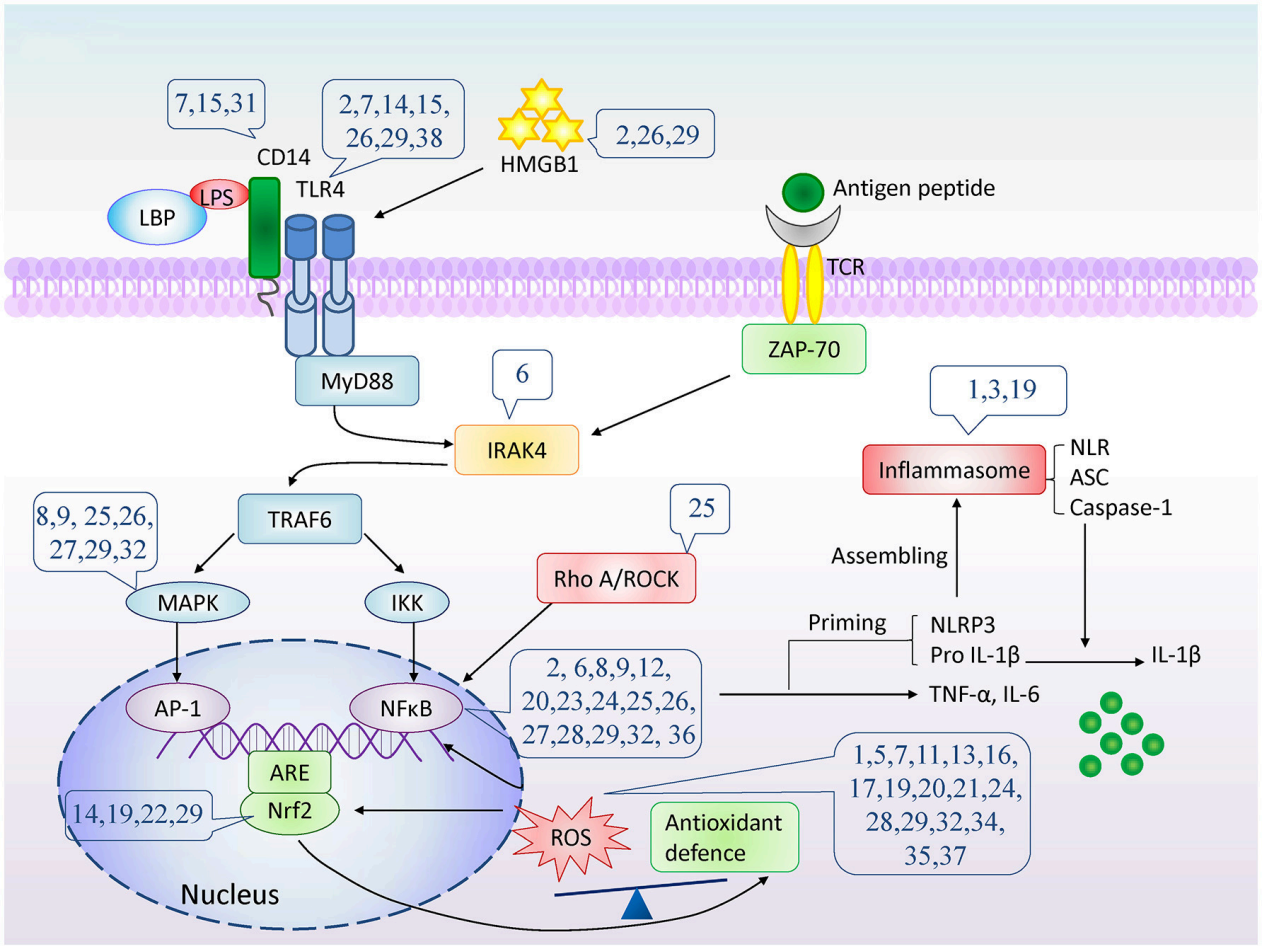
The potential molecular targets of natural compounds inhibition on cytokine/chemokine production. The number indicates compounds described in Table 1. LPS, lipopolysaccharide; LBP, LPS binding protein; TLR4, Toll-like receptor 4; TCR, T-cell receptor; HMGB1, High mobility group box 1; MyD88, myeloid differentiation primary response gene 88; ZAP-70, Zeta-chain-associated protein kinase 70; IRAK4, interleukin-1-receptor associated kinase 4; TNF-α, tumor necrosis factor α; TRAF6, TNF-receptor associated factor 6; MAPK, mitogen-activated protein kinases; IKK, IκB kinase; AP-1, Activator protein 1; NFκB, nuclear factor κB; ARE, antioxidant response element; Nrf2, nuclear factor (erythroid-derived 2)-like 2; RhoA, Ras homolog gene family, member A; ROCK, Rho kinase; ROS, Reactive oxygen species; NLR, NOD-like receptor; NLRP3, NOD-like receptor protein 3; ASC, apoptosis associated spec-like protein containing CARD; IL-1β, interleukin-1β.

The anti-inflammatory effects of natural products are widely reported, which mostly target the inflammatory mediators including cytokines, chemokines, the receptors of cytokine or chemokine, the activated transcription factors, and the additional regulatory factors such as adhesion molecules, nitric oxide, carbon monoxide, and hydrogen sulfide (Kmieć, 2001; Anuar et al., 2006). In this review, the natural compounds with anti-hepatic inflammatory properties are summarized based on their pharmacological mechanisms and described according to their chemical classification (Table 1, Figures 1,2). On the other hand, the clinical evidence from randomized controlled trials (RCTs) (Tables 2, 3) is also presented to visualize the entire profile of the studies on anti-hepatic inflammatory natural products.

**Table 1:** Pharmacological targets of natural products with anti-hepatic inflammatory properties.

**NO**.	**CAS NO**.	Classification^*^	**Compounds**	**Systematic names**	**Related mechanisms**	**Animal models/Cell models**
1	2086-83-1	Alkaloids	Berberine	16,17-dimethoxy-5,7-dioxa-13^5^-azapentacyclo[11.8.0.0^2^,^1^,^0^.0^4^,^8^.0^1^ ^5^,^2^ ^0^]henicosa-1(21),2,4(8),9,13,15,17,19-octaen-13-ylium	Oxidative stress, Inflammasome, P2X7 pathway	Hepatic I/R injury in rat (Sheng et al., 2015)
						Methionine and choline-deficient diet-induced NASH in mouse (Vivoli et al., 2016)
						Acetaminophen-induced liver damage in mouse (Vivoli et al., 2016)
						RAW264.7 cells stimulated by LPS (Vivoli et al., 2016)
						P2X7-knockdown RAW264.7 cells (Vivoli et al., 2016)
2	107-43-7	Alkaloids	Betaine	2-(trimethylazaniumyl)acetate	HMGB1	High-fat diet induced NAFLD in rats (Wu et al., 2011)
					NF-κB	High-fat diet induced NAFLD in rats (Wu et al., 2011)
					TLR4	High-fat diet induced NAFLD in rats (Wu et al., 2011)
						Chronic alcoholic liver injury in rats (Shi et al., 2010)
3	1124-11-4	Alkaloids	Ligustrazine	2,3,5,6-Tetramethylpyrazine	Inflammasome	LO2 cell stimulated by LPS (Zhang et al., 2016)
4	519-02-8	Alkaloids	Matrine	(1R,2R,9S,17S)-7,13-Diazatetracyclo[7.7.1.0^2^,^7^.0^13^,^17^]heptadecan-6-one	Monocyte infiltration	CCl4-induced liver fibrosis in mouse (Shi et al., 2013)
5	518-34-3	Alkaloids	Tetrandrine	9,20,21,25-tetramethoxy-15,30-dimethyl-7,23-dioxa-15,30-diazaheptacyclo[22.6.2.2^3^,6.1^8^,1^2^.1^14^,^18^.0^27^,^31^.0^22^,^33^]hexatriaconta-3,5,8(34),9,11,18,20,22(33),24(32),25,27(31),35-dodecaene	Oxidative stress	Hepatic I/R injury in mouse (Cheng et al., 2008)
6	107-35-7	Amino acid	Taurine	2-aminoethanesulfonic acid	NF-κB; IRAK4	Hepatic I/R injury in rat (Sun et al., 2012)
7	8015-61-0	Quinone—anthraquinone	Aloin	1,8-dihydroxy-3-(hydroxymethyl)-10-[3,4,5-trihydroxy-6-(hydroxymethyl)oxan-2-yl]-10H-anthracen-9-one	TLR4; CD14; Oxidative stress	Chronic alcoholic liver injury in mouse (Cui et al., 2014)
8	518-82-1	Quinone—anthraquinone	Emodin	1,3,8-trihydroxy-6-methylanthracene-9,10-dione	MAPK; NF-κB	Con A-induced hepatitis in mouse (Xue et al., 2015)
						RAW264.7 and EL4 cells stimulated by Con A (Xue et al., 2015)
9	480-44-4	Flavonoids	Acacetin	5,7-dihydroxy-2-(4-methoxyphenyl)chromen-4-one	MAPK; NF-κB	LPS/D-GAlN-induced liver injury in mouse (Cho et al., 2014)
10	491-67-8	Flavonoids	Baicalein	5,6,7-Trihydroxy-2-phenylchromen-4-one	Cytokines; Monocytes infiltration	Con A-induced hepatitis in mouse (Zhang W. et al., 2013)
						LPS-induced liver injury in rat (Chen et al., 2013)
11	21967-41-9	Flavonoids	Baicalin	(2S,3S,4S,5R,6S)-6-(5,6-dihydroxy-4-oxo-2-phenylchromen-7-yl)oxy-3,4,5-trihydroxyoxane-2-carboxylic acid	Oxidative stress	Con A-induced hepatitis in mouse (Liu et al., 2007)
12	446-72-0	Flavonoids	Genistein	5,7-Dihydroxy-3-(4-hydroxyphenyl)chromen-4-one	NF-κB	Alcohol-induced liver fibrosis in rats (Huang et al., 2013)
						LPS/D-GalN-induced hepatitis in mouse (Lin et al., 2014)
13	520-26-3	Flavonoids	Hesperidin	5-hydroxy-2-(3-hydroxy-4-methoxyphenyl)-7-[3,4,5-trihydroxy-6-[(3,4,5-trihydroxy-6-methyloxan-2-yl)oxymethyl]oxan-2-yl]oxy-2,3-dihydrochromen-4-one	Oxidative stress	LPS-induced liver injury in rats (Rotimi et al., 2016)
14	480-11-5	Flavonoids	Oroxylin A	5,7-dihydroxy-6-methoxy-2-phenylchromen-4-one	Nrf2; TLR4	LPS/D-GalN-induced liver injury in mouse (Huang et al., 2015)
15	3681-99-0	Flavonoids	Puerarin	7-hydroxy-3-(4-hydroxyphenyl)-8-[(2S,3R,4R,5S,6R)-3,4,5-trihydroxy-6-(hydroxymethyl)oxan-2-yl]chromen-4-one	TLR2,4; CD14	Chronic alcoholic liver injury in rat induced by Lieber-DeCarli diet (Peng et al., 2013)
16	117-39-5	Flavonoids	Quercetin	2-(3,4-dihydroxyphenyl)-3,5,7-trihydroxychromen-4-one	Oxidative stress	Rat priamry hepatocyte simulated by ethanol (Liu et al., 2010)
17	27740-01-8	Flavonoids	Scutellarin	(2S,3S,4S,5R,6S)-6-[5,6-dihydroxy-2-(4-hydroxyphenyl)-4-oxochromen-7-yl]oxy-3,4,5-trihydroxyoxane-2-carboxylic acid	Oxidative stress	Con A-induced hepatitis in mouse (Tan et al., 2007)
18	632-85-9	Flavonoids	Wogonin	5,7-dihydroxy-8-methoxy-2-phenylchromen-4-one	Monocyte infiltration	LPS-induced liver injury in rat (Chen et al., 2013)
19	51059-44-0	Flavonoids	Wogonoside	5,7-Dihydroxy-8-methoxy-2-phenyl-4H-chromen-4-one	Oxidative stress; Nrf2; Inflammasome	LPS/D-GalN-induced acute liver injury in mouse (Gao et al., 2016)
					Oxidative stress	Hepatic I/R injury in mouse (Tao et al., 2016)
20	23180-57-6	Glucosides	Paeoniflorin	beta-D-Glucopyranoside, (1aS,2R,3aR,5R,5aR,5bS)-5b-((benzoyloxy)methyl)tetrahydro-5-hydroxy-2-methyl-2,5-methano-1H-3,4-dioxacyclobuta(cd)pentalen-1a(2H)-yl	NF-κB	High-cholesterol and high-fat diet—induced NASH in rat (Ma et al., 2016)Con A-induced hepatitis in mouse (Chen M. et al., 2015)
					Cytokines/chemokines; Leukocyte infiltration	Con A-induced hepatitis in mouse (Chen M. et al., 2015)
					Oxidative stress	Hepatic I / R injury in mouse (Tao et al., 2016)
21	75829-43-5	Glucosides	Pinocembrin-7-O-β-D-glucoside	(2S)-5-hydroxy-2-phenyl-7-[(2S,4S,5S)-3,4,5-trihydroxy-6-(hydroxymethyl)oxan-2-yl]oxy-2,3-dihydrochromen-4-one	Oxidative stress	Chronic alcoholic liver injury in rat induced by Lieber-DeCarli diet (Cao et al., 2015)
22	79916-77-1	Glucosides—phenylethanoid glycoside	Forsythiaside A	[(2R,3S,4R,5R,6R)-6-[2-(3,4-dihydroxyphenyl)ethoxy]-4,5-dihydroxy-2-[[(2R,3R,4R,5R,6S)-3,4,5-trihydroxy-6-methyloxan-2-yl]oxymethyl]oxan-3-yl] (E)-3-(3,4-dihydroxyphenyl)prop-2-enoate	Nrf2; NF-κB	LPS/D-GalN—induced liver injury in mouse (Pan et al., 2015)
23	10338-51-9	Glucosides—phenylethanoid glycoside	Salidroside	(2R,3S,4S,5R,6R)-2-(hydroxymethyl)-6-[2-(4-hydroxyphenyl)ethoxy]oxane-3,4,5-triol	Cytokines/chemokines; Leukocyte infiltration; NF-κB	Con A-induced hepatitis in mouse (Hu et al., 2014)
24	24512-63-8	Glucosides—iridoid glycosides	Geniposide	methyl (1S)-7-(hydroxymethyl)-1-[(2S,3R,4S,5S,6R)-3,4,5-trihydroxy-6-(hydroxymethyl)oxan-2-yl]oxy-1,4a,5,7a-tetrahydrocyclopenta[c]pyran-4-carboxylate	Oxidative stress	Hepatic I/R injury in mouse (Kim et al., 2013)
25	23800-56-8	Lactones	Pogostone	4-hydroxy-6-methyl-3-(4-methylpentanoyl)pyran-2-one	NF-κB; MAPK.	Endotoxic shock in mice (Li et al., 2014)
26	104-46-1	phenylpropanoids	Anethole	trans-1-methoxy-4-(1-propenyl)benzene	HMGB1; TLR4; MAPK; NF-κB	Hepatic I/R injury in mouse (Cho et al., 2013)
27	486-21-5	Phenylpropanoid—coumarin	Isofraxidin	7-hydroxy-6,8-dimethoxychromen-2-one	NF-κB; MAPK	LPS-induced liver injury in mice (Liu et al., 2015)
28	22888-70-6	Phenylpropanoids—flavonolignans	Silybin	3,5,7-trihydroxy-2-[3-(4-hydroxy-3-methoxyphenyl)-2-(hydroxymethyl)-2,3-dihydro-1,4-benzodioxin-6-yl]-2,3-dihydrochromen-4-one	NF-κB	Con A-induced hepatitis in mouse (Schümann et al., 2003)
					Oxidative stress	Murine microglia and macrophages stimulated by LPS (Shanmugam et al., 2008)
29	458-37-7	Polyphenol	Curcumin	(1E,6E)-1,7-bis(4-hydroxy-3-methoxyphenyl)hepta-1,6-diene-3,5-dione	Cytokines/Chemokines	Con A-induced hepatitis in mouse (Tu et al., 2011)
					HMGB1	Con A-induced hepatitis in mouse (Wang et al., 2012)
						Acute Propionibacterium acnes -induced liver injury in mouse (Gu et al., 2015)
					TLR2, 4, 9	Con A-induced hepatitis in mouse (Tu et al., 2012)
					MAPK	LPS-induced liver failure in mouse (Zhong et al., 2016)
					NF-κB	db/db mouse (Jiménez-Flores et al., 2014)
						LPS-induced liver failure in mouse (Zhong et al., 2016)
					Nrf2	LPS-induced liver failure in mouse (Zhong et al., 2016)
30	35354-74-6	Polyphenol	Honokiol	2-(4-hydroxy-3-prop-2-enylphenyl)-4-prop-2-enylphenol	Cytokines; Oxidative stress	LPS-induced liver injury in mouse (Sulakhiya et al., 2015)
31	501-36-0	Polyphenol	Resveratrol	5-[(E)-2-(4-hydroxyphenyl)ethenyl]benzene-1,3-diol	CD14	NASH in mouse induced by LPS (Kessoku et al., 2016)
32	65497-07-6	Saponins	Esculentoside A	3-O-[b-D-glucopyranosyl-(1,4)-b-D-xylopyranosyl] phytolaccagenin	NF-κB; MAPK	LO2 cells stimulated by CCl4 (Zhang et al., 2014)
					Oxidative stress	LPS/D-GAlN-induced liver injury in mouse (Zhang et al., 2014)
33	22427-39-0	Saponins	Ginsenoside Rg1	(2R,3R,4S,5S,6R)-2-[[(3S,5R,6S,8R,9R,10R,12R, 13R,14R,17S)-3,12-dihydroxy-4,4,8,10,14-pentamethyl-17-[(2S)-6-methyl-2-[(2S,3R,4S,5S,6R)-3,4,5-trihydroxy-6-(hydroxymethyl)oxan-2-yl]oxyhept-5-en-2-yl]-2,3,5,6,7,9,11,12,13,15,16,17-dodecahydro-1H-cyclopenta[a]phenanthren-6-yl]oxy]-6-(hydroxymethyl)oxane-3,4,5-triol	Cytokines/Chemokines	Con A-induced hepatitis in mouse (Cao et al., 2013)
34	1405-86-3	Saponins	Glycyrrhizin	6-[6-carboxy-2-[(11-carboxy-4,4,6a,6b,8a,11,14b-heptamethyl-14-oxo-2,3,4a,5,6,7,8,9,10,12,12a,14a-dodecahydro-1H-picen-3-yl)oxy]-4,5-dihydroxyoxan-3-yl]oxy-3,4,5-trihydroxyoxane-2-carboxylic acid	Oxidative stress	Con A-induced hepatitis in mouse (Tsuruoka et al., 2009)
35	5508-58-7	Terpenoids—diterpenoid	Andrographolide	(3E,4S)-3-[2-[(1R,4aS,5R,6R,8aS)-6-hydroxy-5-(hydroxymethyl)-5,8a-dimethyl-2-methylidene-3,4,4a,6,7,8-hexahydro-1H-naphthalen-1-yl]ethylidene]-4-hydroxyoxolan-2-one	Oxidative stress	Con A-induced hepatitis in mouse (Shi et al., 2012)
36	38748-32-2	Terpenoids—triepoxide	Triptolide	(1S,2S,4S,5S,7R,8R,9S, 11S,13S)-8-hydroxy-1-methyl-7-(propan-2-yl)-3,6,10,16-tetraoxaheptacyclo [11.7.0.0^∧^{2,4}.0^∧^{2,9}.0^∧^{5,7}. 0^∧^{9,11}.0^∧^{14,18}]icos-14(18)-en-17-one	NF-κB	Hepatic I/R injury in mouse (Wu et al., 2011)
37	6902-77-8	Terpenoids—iridoid	Genipin	methyl (1R,4aS,7aS)-1-hydroxy-7-(hydroxymethyl)-1,4a,5,7a-tetrahydrocyclopenta[c]pyran-4-carboxylate	Oxidative stress	Hepatic I/R injury in mouse (Kim et al., 2013)
38	545-47-1	Terpenoids—triterpenoid	Lupeol	(1R,3aR,5aR,5bR,7aR,9S, 11aR,11bR,13aR,13bR)-3a,5a,5b,8,8,11a-hexamethyl-1-prop-1-en-2-yl-1,2,3,4,5,6,7,7a,9,10,11,11b, 12,13,13a,13b-hexadecahydrocyclopenta[a]chrysen-9-ol	TLR4	LPS/D-GAlN-induced hepatic failure in mouse (Kim et al., 2014)

*Compounds are summarized in alphabetical order of classification. The systematic names of compounds were confirmed on PubChem Compound database*.

* *The sub-class information is provided if there's any*.

**Table 2:** Clinical evidence of natural compounds.

**Compounds**	**Liver diseases**	**Research type**
Berberine	NAFLD (Yan et al., 2015)	RCT
Curcumin	NAFLD (Rahmani et al., 2016; Panahi et al., 2017)	RCT
Glycyrrhizin	Hepatitis C (van Rossum et al., 1999; Iino et al., 2001)	RCT
	Hepatitis B (Zhang and Wang, 2002)	RCT
	Hepatocellular carcinoma related to hepatitis C (Arase et al., 1997)	Retrospective study
Matrine	Hepatitis B (Wang et al., 2017)	RCT
Quercetin	Safety (up to 5 g daily) (Lu et al., 2016)	A phase I dose escalation study
	Potential antiviral activity in hepatitis C (Lu et al., 2016)	
Resveratrol	Hepatitis C (Pennisi et al., 2017)	RCT
Silybin	Alcoholic or viral hepatitis (Vailati et al., 1993)	RCT
	NASH (Chan et al., 2017)	RCT
Taurine	Hepatitis C (Hu et al., 2008)	RCT

**Table 3:** Clinical evidence from the traditional Chinese medicine formulas.

**Traditional Chinese medicine formula**	**Containing compounds**	**Clinical use**	**Research type**
Xiao Chai Hu Tang	Baicalein	Hepatitis B (Tajiri et al., 1991)	RCT
	Baicalin		
	Oroxylin A		
	Scutellarin		
	Wogonin		
	Ginsenoside Rg1		
Yin chen hao tang	Emodin	Improve liver function and fibrosis in postoperative biliary atresia (Kobayashi et al., 2001; Tamura et al., 2007)	RCT
	Geniposide		
	Genipin		

## Suppression of cytokines/chemokines secretion and leukocytes infiltration

The cytokines and chemokines are produced by the hepatic macrophages (Kupffer cells, KCs), natural killer (NK) cells, and NKT cells. KCs can release tumor necrosis factor alpha (TNF-α), interleukin (IL)-6, IL-1β, or leukotrienes, which attract T cells and induce apoptosis of hepatocytes and activation of HSCs (Bilzer et al., 2006). NK cells produce interferon gamma (IFN-γ), IL-8 and apoptosis-inducing TNF-related apoptosis ligand and even directly promote hepatocyte death (Dunn et al., 2007). NKT cells are activated by the glycolipid antigens from bacteria (Kinjo et al., 2005) and involved in antiviral defense mechanisms in hepatitis B (Kakimi et al., 2000).

Recruitment of leukocytes consists of rolling on endothelium mediated by selectins, firm attachment to endothelium mediated by integrins, and migration through interendothelial spaces. TNF-α and IL-1 promote the expression of selectins and integrin ligands on endothelium. Chemokines produced by tissue macrophages increase the avidity of integrins for their ligands and promote directional migration of leukocytes. Monocytes are the largest type of leukocytes. Gr1 (hi) monocytes express high levels of C-C chemokine receptor type 2 (CCR2) but lack CX3C chemokine receptor 1 (CX3CR1). In inflammation, Gr1 (hi) monocytes actively enter inflamed tissue and are considered as the precursors for macrophages and dendritic cells. Gr1 (lo) monocytes lack CCR2 but express high levels of CX3CR1 existing in non-inflamed tissues, representing steady-state precursor cells for tissue macrophages (Tacke et al., 2007). CCR2 mediates entry of Gr1 (hi) monocytes into the inflamed tissues (Boring et al., 1997). Enhanced hepatic expression of C-C chemokine ligand 2 (CCL2) contributes to the formation and maintenance of inflammatory infiltration during chronic liver disease (Marra et al., 1998).

Matrine, a kind of alkaloid, was reported to ameliorate the hepatic infiltration of Gr1 (hi) monocytes and the expression of CCL2 in carbon tetrachloride (CCl_4_)-induced liver injury in mouse and directly inhibited the chemotactic activity and production of CCL2 in HSCs *in vivo* (Shi et al., 2013).

The flavonoids, baicalein and wogonin, were reported to reduce cytokines and chemokines in experimental liver injury, as well as monocytes infiltration. Baicalein suppressed serum TNF-α, IFN-γ, hepatic infiltration of monocytes, and up-regulated the apoptosis of monocytes in the liver in concanavalin A (Con A)-induced hepatitis (Zhang Y. et al., 2013). Baicalein and wogonin were both found to attenuate lipopolysaccharide (LPS)-induced liver sinusoidal endothelial cells activation and HSCs migration by down-regulating CCL2 expression (Chen et al., 2013).

The polyphenolic compounds, curcumin and honokiol, were reported to decrease the cytokines, chemokines and infiltration of T cells. Curcumin suppressed the production of TNF-α, IFN-γ, and IL-4, infiltration of CD4 (+) T cells and the expression of intercellular adhesion molecule-1 (ICAM-1) and the interferon-inducible chemokine, C-X-C motif chemokine 10 (CXCL10), in hepatic tissue (Tu et al., 2011). Honokiol ameliorated liver damage and levels of IL-1β, IL-6, and TNF-α in serum or liver tissue in LPS or LPS combined with D-galactosamine (LPS/D-GalN) challenged mice (Sulakhiya et al., 2015).

Ginsenoside Rg1, a kind of saponin, was reported to suppress pro-inflammatory cytokines, the expression of ICAM-1 and CXCL10 in hepatic tissue in Con A-induced hepatitis (Cao et al., 2013).

## Action on pattern recognition receptors pathways

The pathogen-associated molecular patterns (PAMPs) and damage-associated molecular patterns (DAMPs) are recognized by pattern recognition receptors expressed on the target cells including phagocytes, dendritic cells, epithelial cells, and many other cells. Inflammasomes and Toll-like receptors (TLRs) are the two most important pattern recognition receptor families.

### Inactivation of inflammasome pathway

Inflammasome is a cytoplasmic complex composed of NOD-like receptor (NLR), the adapter, apoptosis associated spec-like protein containing CARD (ASC), and the effecter, caspase-1 protein (Martinon et al., 2002). Several members of inflammasomes family have been identified (Negash and Gale, 2015). NOD-like receptor protein 3 (NLRP3) is the most thoroughly studied to date.

Inflammasome activation requires priming and assembly activating steps to mediate both IL-1β and IL-18 production. The priming step triggered by PAMP or DAMP recognition, up-regulates NLR protein and initiates inactive proIL-1β and proIL-18 production. The assembly activating step drives inflammasome components to associate and form a macromolecular complex that mediates active caspase-1 production and subsequent maturation and secretion of IL-1β and IL-18. Inflammasomes can be induced and activated in hepatocytes, HSCs (Masumoto et al., 2001; Watanabe et al., 2009), the sinusoidal endothelial cells (Masumoto et al., 2001; Imaeda et al., 2009), and fibroblasts (Rawat et al., 2010). KCs robustly activate the NLRP3 inflammasome to produce high levels of IL-1β (Negash et al., 2013).

The alkaloids, berberine and ligustrazine, were found to inhibit the NLRP3 inflammasome. Berberine inhibited hepatic necroinflammation, IL-1β, and NLRP3 inflammasome in non-alcoholic steatohepatitis (NASH) induced by methionine and choline - deficient diet in mouse, which based on interference with activation of P2x7, a purinergic receptor involved in inflammasome activation (Vivoli et al., 2016). Ligustrazine was found to reduce NLRP3 and cleaved-caspase-1, to decrease IL-1β cleavage, and IL-1β secretion in human LO2 hepatocytes stimulated by LPS (Zhang et al., 2016).

The flavonoid, wogonoside, inhibited liver injury and the expression of hepatic NLRP3, ASC, caspase-1, and IL-1β induced by LPS/D-GalN in mice (Gao et al., 2016).

### Inhibition of TLRs pathway

#### Prevention on HMGB1 release

High mobility group box 1 (HMGB1) is one of the first identified members of the DAMP molecular family (Yang et al., 2015). HMGB1 release occurs during tissue injury or microbial invasion via passive and active ways. Passive release is nearly instantaneous, which occurs in the context of necrotic cell death. The active HMGB1 secretion depends on acetylation of nuclear localization sequences sites, which prevents the continuous bidirectional shuttle of HMGB1 between the cytoplasm and the nucleus, and leads to cytoplasmic accumulation of hyperacetylated HMGB1. Caspase-1 activated by the inflammasome system is required in pyroptosis, a gradual induction of programmed, proinflammatory cell death, which allows cytoplasmic HMGB1 to reach the extracellular space (Lamkanfi et al., 2010; Lu et al., 2012). HMGB1 can interact with TLR2, TLR4, TLR9, and the receptor for advanced glycation endproducts, in which, TLR4 is the dominant one (Andersson and Tracey, 2011). HMGB1 binds to TLR4 and activates macrophages (Yang et al., 2010) through nuclear factor κB (NF-κB) pathway (Park et al., 2004). HMGB1 is critical for neutrophil recruitment, injury amplification, and lethal liver injury (Huebener et al., 2015).

The alkaloid, betaine, decreased serum levels of alanine aminotransferase (ALT), aspartate aminotransferase (AST) and histological scores for steatosis, inflammation, and necrosis, as well as serum and hepatic HMGB1 in non-alcoholic fatty liver disease (NAFLD) induced by high-fat diet in rats (Zhang W. et al., 2013).

Anethole, a compound of phenylpropanoids, was found to attenuate liver injury and pro-inflammatory cytokines secretion in hepatic ischemia/reperfusion (I/R) mouse, and to inhibit the release of HMGB1 by prevention on nuclear translocation of interferon regulatory factor and interaction to histone acetyltransferase p300 (Cho et al., 2013).

The polyphenolic compound, curcumin, was reported to decrease serum ALT, TNF-α, IFN-γ, and hepatic necrosis and apoptosis in propionibacterium acnes-induced liver injury (Gu et al., 2015) and Con A-induced hepatitis (Wang et al., 2012), which related to its inhibition on HMGB1 cytoplasmic translocation and expression by down-regulation of acetylation of lysine.

#### Down-regulation of TLR4 and CD14 expression

TLRs, especially TLR4, are receptors of LPS, the component of outer membrane of Gram-negative bacteria. The gut-derived LPS is involved in the pathogenesis of inflammation in several kinds of liver diseases, such as NAFLD and alcoholic liver disease. LPS is recognized by the complex of CD14, TLR4, and myeloid differentiated protein-2 (Fujihara et al., 2003). A serum LPS binding protein (LBP) transfers LPS to CD14. CD14 concentrates LPS and presents it to TLR4 to activate the down-stream signaling cascade and ultimately initiate transcription of pro-inflammatory factors. Leptin was recently found to induce CD14 expression *via* activation of signal transducer and activator of transcription 3 (STAT3) signaling in KCs, resulting in enhanced responsivity against low-dose LPS in the liver (Imajo et al., 2012), which contributes to the progression of NAFLD.

The alkaloid, betaine, resulted in significant amelioration of serum ALT, AST, endotoxin, TNF-α, IFN-γ, and IL-18 and histology in liver, as well as down-regulation of the expression of hepatic TLR4 mRNA and protein in chronic alcoholic liver injury induced by high fat diet plus ethanol and fish oil in rats (Shi et al., 2010).

The flavonoids, oroxylin A and puerarin, were found to down-regulate the expression of TLR4 and CD14. Oroxylin A inhibited hepatic TLR4 expression and the downstream NF-κB activation in LPS/D-GalN-induced liver injury (Huang et al., 2015). Puerarin decreased hepatic inflammation in chronic alcohol-intake rats, and inhibited the protein expression of CD14, TLR2, and TLR4 (Peng et al., 2013).

The polyphenolic compounds, resveratrol and curcumin were reported to be effective on CD14 and TLR4. Resveratrol dramatically inhibited inflammation in a low-dose LPS-induced model of NASH through inhibition of the STAT3-CD14 pathway in KCs (Kessoku et al., 2016). Curcumin also reduced hepatic TLR2, TLR4, and TLR9 in Con A-stimulated liver tissues in mouse (Tu et al., 2012).

Aloin, a compound of anthraquinone, reduced liver injury in alcoholic hepatitis mice, and simultaneously, decreased serum LPS and the protein expression of hepatic TNF-α, TLR4, and MyD88 (Cui et al., 2014).

#### Inactivation of TLR4-downstream signaling

The downstream signaling of TLR4 involves myeloid differentiation primary response gene 88 (MyD88)-dependent and MyD88-independent pathway. In MyD88-dependent pathway, MyD88 associates with TLR and IL-1 receptor and recruits interleukin-1-receptor associated kinase (IRAK) to the receptor complex. IRAKs are subsequently phosphorylated and dissociated from the receptor complex and interact with TNF-receptor associated factor 6 (TRAF6) (Li and Verma, 2002). TRAF6 activates mitogen-activated protein kinases (MAPK) and NF-κB pathway, and initiates pro-inflammatory genes expression.

The alkaloid, betaine, ameliorated hepatitis and decreased hepatic mRNA and protein levels of TLR4 and NF-κB in NAFLD rats induced by high-fat diet (Zhang W. et al., 2013).

The flavonoid, acacetin, attenuated serum TNF-α and IL-6 levels, and down-regulated protein expression of TLR4, activation of p38 MAPK/JNK and NF-κB nuclear translocation in D-GaIN-challenged mice (Cho et al., 2014).

Pogostone, a compound of lactones, reduced liver injury and mortality induced by LPS in mice by inhibition on phosphorylation of p38 MAPK and NF-κB (Li et al., 2014).

The phenylpropanoids, anethole and isofraxidin, were found to inactivate the TLR4-downstream signaling. Anethole attenuated liver inflammation in hepatic I/R mice and down-regulate the protein expression of TLR4, MyD88, and activation of MAPK and NF-κB (Cho et al., 2013). Isofraxidin, a compound of coumarin, reduced LPS-induced hepatic injury, phosphorylation of extracellular signal-regulated kinases 1/2 (ERK1/2), c-Jun N-terminal kinase (JNK) and p38 MAPKs, and NF-κB activation simultaneously (Liu et al., 2015).

The polyphenolic compound, curcumin, was reported to inhibit the activation of p38 MAPK/JNK cascade, which correlated to its amelioration on LPS-induced liver injury (Zhong et al., 2016).

The anthraquinone, emodin, decreased Con A-induced hepatic necrosis, pro-inflammatory cytokines and chemokines, and CD4 (+) and F4/80 (+) cells infiltration in the liver, accompanied with the inactivation of p38 MAPK and NF-κB *in vivo* and *in vitro* (Xue et al., 2015).

Esculentoside A, a compound of saponins, reduced liver injury, F4/80 (+) and CD11b (+) cells infiltration and activation of NF-κB and MAPK in the liver stimulated by CCl_4_ (Zhang et al., 2014).

The terpenoid, lupeol, reduced liver injury, the protein expression of TLR4, MyD88, IRAK-1 and TRAF6, and NF-κB nuclear translocation in LPS/D-GaIN-induced fulminant hepatic failure in mice (Kim et al., 2014).

## Inactivation of NF-κB

Besides TLRs signal, NF-κB activation is also mediated by TNF-α, T-cell receptor signaling (Li and Verma, 2002), and RhoA/Rho kinase (ROCK) (Perona et al., 1997).

IRAK-4, a member of the IRAK family, functions upstream of the other IRAKs and involves in signaling of innate immune responses from TLRs and T-cell receptors.

Taurine, a sulfur-containing β-amino acid, the major constituent of bile, protected against hepatic I/R injury and inhibited TNF-α expression in KCs partially by down-regulation of IRAK-4 and the downstream NF-κB activation (Sun et al., 2012).

The flavonoid, genistein, decreased levels of inflammation mediators, including IL-6, TNF-α by inactivation of NF-κB in alcohol-induced liver fibrosis in rats (Huang et al., 2013; Chen S. R. et al., 2015) and LPS/D-GalN-induced hepatitis in mouse (Lin et al., 2014).

The glucosides, paeoniflorin and salidroside, were found to inactivate NF-κB. Paeoniflorin ameliorated liver injury and inhibited serum TNF-α in NASH in rats and simultaneously inhibited the activity of ROCK and activation of NF-κB in liver (Ma et al., 2016). Paeoniflorin was also reported to inhibit the activation of NF-κB in liver tissue in Con A-induced hepatitis (Chen M. et al., 2015). Salidroside (Hu et al., 2014) inhibited Con A-induced hepatits, proinflammatory cytokines, hepatic infiltration of CD4 (+), CD8 (+) by regulating interferon-inducible CXCL10 and NF-κB activation in liver tissue (Hu et al., 2014).

The polyphenol, curcumin, down-regulated the protein expression of NF-κB in the liver of db/db mouse (Jiménez-Flores et al., 2014).

Silybin, a compound of flavonolignans, reduced plasma levels of transaminases and liver content of pro-inflammatory cytokines, inhibited hepatic NF-κB activation, and increased plasma and tissue levels of IL-10 in hepatitis induced by Con A in mouse (Schümann et al., 2003).

The terpenoid, triptolide suppressed neutrophil infiltration, pro-inflammatory cytokine level and NF-κB activation in I/R liver in mice (Wu et al., 2011).

## Inhibition of ROS production

Reactive oxygen species (ROS) induces chronic inflammation by the induction of cyclooxygenase-2, inflammatory cytokines (TNF-α, IL-1, IL-6), chemokines (IL-8, chemokine receptor type 4), and pro-inflammatory transcription factors (NF-κB) (Gupta et al., 2012). The widely studied and understood ROS family includes the superoxide anion, hydroxyl radical, hydrogen peroxide, and hypochlorous acid (Thannickal and Fanburg, 2000). ROS can rapidly combine with nitric oxide to form reactive nitrogen species (Beckman, 1996), which induces nitrosative stress and contributes to the pro-inflammatory burden of ROS. Superoxide dismutase (SOD), catalase, glutathione peroxidase (GPx), and peroxiredoxins are important enzymes involved in antioxidant reactions (Mittal et al., 2014). Glutathione (GSH) is a very powerful endogenous antioxidant. Synthesis of GSH is regulated by catalytic and modifier subunit of glutamate-cysteine ligase, which are characteristic target genes of nuclear factor (erythroid-derived 2)-like 2 (Nrf2) (Solis et al., 2002).

The alkaloids, berberine and tetrandrine, were reported to target the production of ROS. Berberine dramatically attenuated the hepatic histopathologic damage, restored the liver function, and decreased the oxidative stress level in I/R liver in rat (Sheng et al., 2015). Tetrandrine ameliorated I/R liver injury by suppressing oxidative stress, including decreasing malondialdehyde (MDA), myeloperoxidase (MPO), and increasing SOD (Cheng et al., 2008).

The flavonoids, including scutellarin, baicalin, hesperidin, quercetin, wogonoside, and oroxylin A, were reported to inhibit ROS. Scutellarin (Tan et al., 2007) decreased the production of ROS and the expression of inducible nitric oxide synthase (iNOS) in Con A-induced hepatitis. Baicalin (Liu et al., 2007) reduced MPO activity and lipid peroxidation, and increased the anti-oxidative SOD expression in liver tissue in Con A-injected mice. Hesperidin down-regulated the expression of nitric oxide, hydroperoxides, and thiobarbituric acid reactive substances, and increased GSH, glutathione reductase, GPx and glutathione S-transferases (GST) in the liver of rats treated with LPS (Rotimi et al., 2016). Quercetin reduced secretion of AST, MDA, and increased levels of GSH and SOD in rat primary hepatocytes stimulated by ethanol (Liu et al., 2010). Wogonoside (Gao et al., 2016) decreased inflammatory factors accompanied with inhibition on the production of MDA by activating Nrf2 and increasing heme oxygenase-1 (HO-1) and catalytic subunit of glutamate-cysteine ligase in LPS/D-GalN-induced liver injury.

Silybin, a compound of flavonolignans, attenuated iNOS and TNF-α expression in macrophages induced by LPS *in vitro* (Shanmugam et al., 2008).

The glucosides including paeoniflorin, geniposide, pinocembrin-7-O-β-D-glucoside and forsythiaside A, were found to inhibit ROS. Paeoniflorin (Tao et al., 2016) ameliorated I/R liver injury by decreasing MDA content and enhancement of the activities of hepatic SOD, GSH, and GPx. Geniposide (Kim et al., 2013) ameliorated I / R liver injury by decreasing MDA and increasing the ratio of GSH / glutathione disulfide and the protein expression of HO-1 in liver. In chronic ethanol-challenged mouse, pinocembrin-7-O-β-D-glucoside significantly reduced hepatic ROS and MDA, and restored the activity of GSH, SOD, and GPx and increased the hepatic expression of Nrf2 and the downstream anti-oxidant HO-1 (Cao et al., 2015). Forsythiaside A (Pan et al., 2015) protected against LPS/D-GalN-induced liver injury by up-regulated the expression of Nrf2 and HO-1 in the liver.

The polyphenolic compound, curcumin, was reported to reduce hepatic oxidative stress in Con A-induced hepatitis (Wang et al., 2012), to decrease the levels of MDA and 4-hydroxy nonyl alcohol in the liver of NASH rats (Wang et al., 2015) and to accelerate liver antioxidant enzymes levels, including SOD, catalase, GSH and GPx, in LPS-induced liver injury (Zhong et al., 2016).

The saponnins, glycyrrhizin and esculentoside A, inhibited the production of ROS. glycyrrhizin (Tsuruoka et al., 2009) reduced hepatitis in Con A-stimulated mice and inhibited the expression of iNOS. Esculentoside A (Zhang et al., 2014) inhibited MDA release and increased GPx activity in liver induced by CCl4 and LPS/D-GalN.

## Clinical evidence

The most powerful evidence of a drug candidate comes from RCTs. In this part, the natural compounds verified in RCTs are summarized.

The alkaloids, matrine and berberine, were verified in RCTs. Matrine combined with INF exhibited better clinical efficacy including negative conversion rate of hepatitis B virus e-antigen, hepatitis B virus DNA, and AST level, and fewer adverse effects than did INF monotherapy in patients with chronic hepatitis B (Wang et al., 2017). Berberine improved serum ALT, AST, hepatic fat content, and insulin resistance in NAFLD patients (Yan et al., 2015; Wei et al., 2016).

Taurine, the sulfur-containing β-amino acid, was reported to decrease serum ALT and AST activities and levels of cholesterol and triglyceride in hepatitis C patients (Hu et al., 2008).

The flavonoid, quercetin, exhibited safety (up to 5 g daily) and a potential for antiviral activity in some hepatitis C patients in a phase I dose escalation study (Lu et al., 2016).

Silybin, a compound of flavonolignans, has also been reported to improve liver enzymes in alcoholic or viral hepatitis (Vailati et al., 1993). In a randomized trial on NASH, patients in silybin group had reductions in fibrosis based on histology and AST than did the placebo group (Chan et al., 2017).

The polyphenols, resveratrol and curcumin, were verified in RCTs. Resveratrol combined with pegylated-INF-α2b and Ribavirin was reported to improve AST, viremia, histological activity index and C-reactive protein in hepatitis C patients comparing to that in pegylated-INF-α2b, Ribavirin and placebo group (Pennisi et al., 2017). Curcumin significantly reduced the liver fat content, serum levels of ALT, AST, total cholesterol, triglycerides and glucose in NAFLD patients compared with the placebo group (Rahmani et al., 2016; Panahi et al., 2017).

The saponin, glycyrrhizin, is used intravenously or orally in chronic hepatitis B and C patients. And its preparation under the name of Stronger Neo-Minophagen C decreased ALT and AST levels in patients with chronic hepatitis in multiple double-blind studies (van Rossum et al., 1999; Iino et al., 2001; Zhang and Wang, 2002). It was suggested that glycyrrhizin had a preventive effect on the development of hepatocellular carcinoma in patients with chronic hepatitis C (Arase et al., 1997).

It's interesting that the effects of curcumin and resveratrol, the two controversial polyphenolic compounds, were both supported by the results of RCTs. Curcumin targets multiple anti-inflammatory mechanisms, which indicates that curcumin is probably a powerful anti-inflammatory compound. But recently, the effects of curcumin were questioned because it was classified as a candidate of panassay interference compounds and invalid metabolic panaceas (Nelson et al., 2017). Although the RCTs on many diseases based on its anti-inflammatory effects have been conducted (Derosa et al., 2016; Sahebkar et al., 2016), there was rare report about the effect of curcumin on human hepatitis under RCT design until the reports of Panahi et al. and Rahmani et al. come out. It is encouraging even if the histological evidence were absent. The anti-inflammatory potential of resveratrol was demonstrated in metabolic syndrome in animal studies, while the results from trials in metabolic syndrome were not as promising as the pre-clinical data. It was also reported that resveratrol did not decrease the levels of ALT and AST, as well as the insulin resistance and steatosis in NAFLD patients compared with baseline (Chachay et al., 2014). To confirm the effects of resveratrol, more RCTs designed with histological checkpoints needed to be developed.

On the other hand, several natural products with anti-hepatic inflammatory properties are the predominant active compounds in the traditional Chinese formulas, Xiao Chai Hu Tang (in Kampo name of Sho-saiko-to) and Yin chen hao tang (in Kampo name of Inchin-ko-to) which are popular medicine used to treat liver diseases in ancient China and have been verified in RCTs up to now. Xiao Chai Hu Tang [containing baicalin, baicalein, scutellarin (Shimizu, 2000), oroxylin A (Liu et al., 2002), wogonin and ginsenoside Rg1(Ohtake et al., 2004)] was reported to increase INFγ and hepatitis B virus core antibody and hepatitis B virus e-antibody in peripheral blood mononuclear cells from patients of chronic hepatitis B (Kakumu et al., 1991), which partly contributed to its promotion on clearance of hepatitis B virus e-antigen in the children with chronic hepatitis B (Tajiri et al., 1991) and to adjust the decreased IL-10 production and the increased IL-4 and IL-5 production of mononuclear cells from patients with hepatitis C (Yamashiki et al., 1997). Yin chen hao tang [containing geniposide, genipin (Inao et al., 2004), and emodin (Imanishi et al., 2004)] was reported to improve serum ALT, AST, gamma-glutamyl transferase, and fibrosis indicators (Hyaluronic acid, type III procollagen N paptide, and type IV collagen) in postoperative biliary atresia patients (Kobayashi et al., 2001; Tamura et al., 2007).

## Concluding remarks

The anti-inflammatory effects of natural compounds in liver have been widely demonstrated in different models of liver injury *in vitro* and *in vivo*. It has been demonstrated that the inflammation mediators including cytokines, chemokines, pattern recognition receptors, the activated transcriptional factors and the regulatory factor, ROS, are the potential targets of these compounds. The anti-hepatic inflammatory activity is not limited to certain class of compounds, since the alkaloids, quinine, flavonoids, glucosides, phenylpropanoids, polyphenols, sapoins, terpenoids, etc., all present activity on hepatitis. It is also obvious that the flavonoids are the majority of these compounds and mostly target the inhibition of oxidative stress.

But, most of the basic researches are limited to observations of the changes of inflammation parameters and the relative pathways. Only berberine was confirmed to inhibit inflammasome by inactivation of P2x7 by using P2x7-knockdown cell line. To identify the pharmacological targets, it is necessary to employ the transgenic models of the potential target molecules in studies in the future. On the other hand, using the ideal animal models with the characters of human hepatitis in pre-clinic state probably prevent the controversial results from the clinical trials. For example, con A-induced acute hepatitis by a massive cytokine storm is not a reliable animal model of autoimmune hepatitis which is characterized by persistent chronic inflammation. While, the cytochrome P450 2D6 (CYP2D6) humanized mouse targeting the human autogantigen CYP2D6 is a valid model to study autoimmune mediated liver damage (Christen et al., 2007).

In most of the RCTs, ALT and AST were measured as hallmark of hepatitis. The histological evidence was only provided in few clinical trials, such as the RCTs of silybin and resveratrol. Since liver biopsy is still the golden diagnosis standard for the most types of hepatitis, the histological examination should be conducted to confirm the effects of natural compounds on inflammation in liver tissue.

Finally, as we see in the clinical evidence, the traditional Chinese medicine formulas which contain groups of compounds present positive results on hepatitis. It seems like a potential strategy for hepatitis treatment to develop new compound formulas consisting of natural compounds with clear chemical structures and action targets, since the pathogenesis of hepatitis is more like an orchestra of pathological mechanisms instead of single target.

The available evidence from basic and clinical research suggest the natural compounds with anti-hepatic inflammatory properties are potential resource for new drug development for liver diseases. The underlying mechanisms and safety are deserved to be investigated thoroughly by optimized animal and clinical studies.

## Author contributions

JP conceived and designed the project and wrote the manuscript.

### Conflict of interest statement

The author declares that the research was conducted in the absence of any commercial or financial relationships that could be construed as a potential conflict of interest.

## Abbreviations

HSC: hepatic stellate cell
RCT: randomized controlled trial
KC: Kupffer cell
NK: natural killer cell
TNF-α: tumor necrosis factor alpha
IL: interleukin
IFN-γ: interferon gamma
CCR2: C-C chemokine receptor type 2
CX3CR1: CX3C chemokine receptor 1
CCL2: C-C chemokine ligand 2
Con A: concanavalin A
CCl_4_: tetrachloride
LPS: lipopolysaccharide
ICAM-1: intercellular adhesion molecule-1
CXCL10: C-X-C motif chemokine 10
D-GalN: D-galactosamine
PAMP: pathogen-associated molecular pattern
DAMP: damage-associated molecular pattern
TLR: Toll-like receptor
NLR: NOD-like receptor
ASC: apoptosis associated spec-like protein containing CARD
NLRP3: NOD-like receptor protein 3
NASH: non-alcoholic steatohepatitis
HMGB1: high mobility group box 1
NF-κB: nuclear factor κB
ALT: alanine aminotransferase
AST: aspartate aminotransferase
NAFLD: non-alcoholic fatty liver disease
I/R: ischemia/reperfusion
LBP: lipopolysaccharide binding protein
STAT3: signal transducer and activator of transcription 3
MyD88: myeloid differentiation primary response gene 88
IRAK: interleukin-1-receptor associated kinase
TRAF6: TNF-receptor associated factor 6
MAPK: mitogen-activated protein kinases
ERK1/2: extracellular signal-regulated kinases 1/2
JNK: c-Jun N-terminal kinase
ROCK: Rho kinase
ROS: Reactive oxygen species
SOD: superoxide dismutase
GPx: glutathione peroxidase
GSH: glutathione
Nrf2: nuclear factor (erythroid-derived 2)-like 2
MDA: malondialdehyde
MPO: myeloperoxidase
iNOS: inducible nitric oxide synthase
GST: glutathione S-transferases
HO-1: heme oxygenase-1
CYP2D6: cytochrome P450 2D6.
